# Idiopathic pyloric stenosis

**DOI:** 10.11604/pamj.2016.25.23.10707

**Published:** 2016-09-27

**Authors:** Dimitrios Manatakis, Maria Sioula

**Affiliations:** 1 Department of Surgery, “Konstantopouleio” General Hospital, Nea Ionia, Athens, Greece

**Keywords:** Pyloric stenosis idiopathic, adult

## Image in medicine

Idiopathic pyloric stenosis in adults is a rare condition of unknown etiology, caused by hypertrophy and hyperplasia of the pyloric musculature with gastric outlet obstruction and delayed gastric emptying. It should be differentiated from the secondary form, caused by recurrent peptic ulcers, malignancy or hypertrophic gastritis. Symptoms include epigastric pain, early satiety and postprandial nausea and vomiting, however many cases remain asymptomatic for years. Although pyloroplasty is an acceptable option, distal gastric resection is the recommended approach, to avoid recurrence. We report the case of 44-year-old man who presented at the emergency department, with a 5-day history of protracted vomiting, abdominal pain and constipation. He appeared severely malnourished, complaining of malaise and generalized weakness. Clinical examination revealed hypotension (90/60 mmHg) and tachycardia (100 bpm), abdominal distention with hyper-resonance during percussion and diffuse tenderness on palpation. Other than leukocytosis (12,000/mcL), laboratory tests were within normal range and the nasogastric tube produced 200ml of clear, non-bilious fluid. CT scans showed a markedly dilated stomach (A), causing compression of the inferior vena cava and the solid organs of the upper abdomen (B,C). He was transferred to the operating theater for exploratory laparotomy, however he collapsed hemodynamically upon induction to anesthesia, and succumbed despite attempts to CPR.

**Figure 1:**
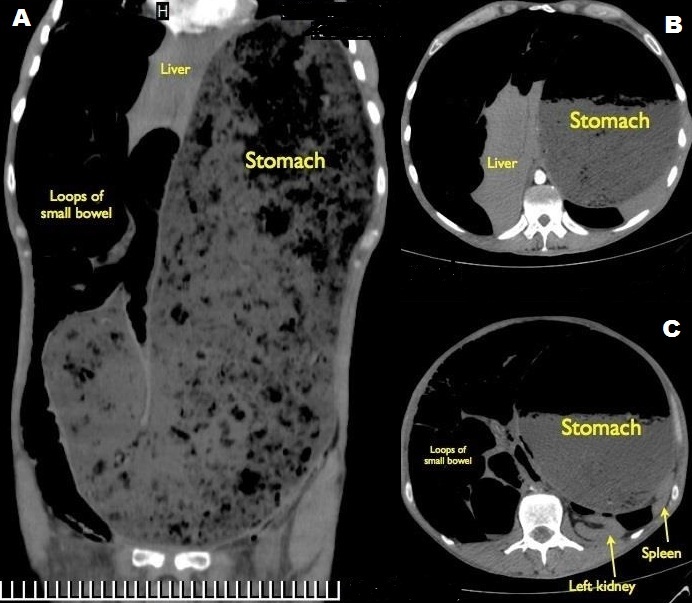
Idiopathic pyloric stenosis

